# Multifactorial Modes of Action of Arsenic Trioxide in Cancer Cells as Analyzed by Classical and Network Pharmacology

**DOI:** 10.3389/fphar.2018.00143

**Published:** 2018-02-27

**Authors:** Mona Dawood, Sami Hamdoun, Thomas Efferth

**Affiliations:** Department of Pharmaceutical Biology, Institute of Pharmacy and Biochemistry, Johannes Gutenberg University, Mainz, Germany

**Keywords:** arsenic trioxide, drug resistance, pharmacogenomics, AP-1, NF-κB

## Abstract

Arsenic trioxide is a traditional remedy in Chinese Medicine since ages. Nowadays, it is clinically used to treat acute promyelocytic leukemia (APL) by targeting PML/RARA. However, the drug’s activity is broader and the mechanisms of action in other tumor types remain unclear. In this study, we investigated molecular modes of action by classical and network pharmacological approaches. CEM/ADR5000 resistance leukemic cells were similar sensitive to As_2_O_3_ as their wild-type counterpart CCRF-CEM (resistance ratio: 1.88). Drug-resistant U87.MG ΔEGFR glioblastoma cells harboring mutated epidermal growth factor receptor were even more sensitive (collateral sensitive) than wild-type U87.MG cells (resistance ratio: 0.33). HCT-116 colon carcinoma p53^-/-^ knockout cells were 7.16-fold resistant toward As_2_O_3_ compared to wild-type cells. Forty genes determining cellular responsiveness to As_2_O_3_ were identified by microarray and COMPARE analyses in 58 cell lines of the NCI panel. Hierarchical cluster analysis-based heat mapping revealed significant differences between As_2_O_3_ sensitive cell lines and resistant cell lines with *p*-value: 1.86 × 10^-5^. The genes were subjected to Galaxy Cistrome gene promoter transcription factor analysis to predict the binding of transcription factors. We have exemplarily chosen NF-kB and AP-1, and indeed As_2_O_3_ dose-dependently inhibited the promoter activity of these two transcription factors in reporter cell lines. Furthermore, the genes identified here and those published in the literature were assembled and subjected to Ingenuity Pathway Analysis for comprehensive network pharmacological approaches that included all known factors of resistance of tumor cells to As_2_O_3_. In addition to pathways related to the anticancer effects of As_2_O_3_, several neurological pathways were identified. As arsenic is well-known to exert neurotoxicity, these pathways might account for neurological side effects. In conclusion, the activity of As_2_O_3_ is not restricted to acute promyelocytic leukemia. In addition to PML/RARA, numerous other genes belonging to diverse functional classes may also contribute to its cytotoxicity. Network pharmacology is suited to unravel the multifactorial modes of action of As_2_O_3_.

## Introduction

Cancer represents a major cause of mortality. It accounted for about 7 Mio deaths in 2000 (Guilbert, 2001) and 8.2 million deaths in 2012 worldwide (Ferlay et al., 2015). In 2008, 12.7 Mio new cancer cases were estimated (Jemal et al., 2010). Recently, 1,688,780 new cancer cases and about 600,920 cancer deaths were estimated to occur in the United States (Siegel et al., 2017). Although, significant progress has been made in cancer therapy during the past years, treatment outcome is still largely hampered by drug resistance and severe side effects (Wiench et al., 2012). Various mechanisms of anticancer drug resistance have been described, e.g., point mutations in drug targets, drug efflux pumps, xenobiotic detoxification mechanisms, resistance to apoptosis, repair DNA damage, alteration of cell proliferation features and others (Cree and Charlton, 2017). For this reason, novel compounds are urgently required for the improvement of treatment outcomes and survival prognosis of patients (Wiench et al., 2012).

Arsenic is a semimetal naturally existing in earth, water and air. It is found in many chemical forms such as yellow arsenic (As_2_S_3_), red arsenic (As_2_S_2_), white arsenic (As_2_O_3_) etc. (Miller et al., 2002). Since ages, As_2_O_3_ is used in traditional Chinese medicine. In the 1970s, Chinese scientists tested the potential of As_2_O_3_ to treat acute PML (Rao et al., 2013). Today, As_2_O_3_ is clinically used worldwide to treat APL and multiple myeloma (Soignet et al., 1998; Breccia and Lo-Coco, 2012; Iland et al., 2014). A major driver of tumorigenesis of APL is the *PML/RARA* oncogene. Interestingly, As_2_O_3_ targets this oncogene, which explains its strong activity in this tumor entity. In combination with retinoic acid, As_2_O_3_ can cure up to 90% of APL patients (Lallemand-Breitenbach et al., 2012). After binding of As_2_O_3_ to PML/RARA, sumoylation and ubiquitination takes place. Further events included in As_2_O_3_’s modes of action include reactive oxygen species generation, inducing apoptosis and cell cycle arrest, activating caspases 8 and 9, down-regulating VEGF thus suppress the angiogenesis as well as inhibition of tumor invasion and metastasis (Zhao et al., 1997; Huang et al., 1999; Perkins et al., 2000; Roboz et al., 2000; Anderson et al., 2002; Hayashi et al., 2002; Liu et al., 2003).

Independent from the activity of As_2_O_3_ in APL, other tumor types may also be affected via other mechanisms, e.g., the hedgehog signaling pathway in medulloblastoma (Beauchamp and Uren, 2012; Klinger et al., 2017), indicating that the full range of mechanisms of As_2_O_3_ has not been elucidated yet.

Recently, network pharmacology becomes an important bioinformatics tools for identifying the mechanism of action of traditional Chinese medicine (TCM). Several methodologies including proteomics, metabolomics, genomics and serum pharmacokinetics are used to identify molecular target and mechanisms of TCM formulas. Applying this methods will lead to a shift from one drug- one target model to network target-multi-components models (Liang et al., 2014). In addition, applying network analysis (protein–protein interaction) may identify drug-target-related proteins (Li and Zhang, 2013).

In an endeavor to study the cytotoxic activity of As_2_O_3_ in cell lines of other tumor types than APL and to identify possible novel modes of action, we undertook the present project. The aims of this study were firstly to investigate whether classical drug resistance mechanisms such as *P*-glycoprotein, the tumor suppressor p53 and the EGFR may decrease cellular responsiveness to As_2_O_3_. The anti-proliferation activity of As_2_O_3_ and predominantly its effects against colon carcinoma resistant cell lines HCT116 (p53^-/-^), U87M. ΔEGFR glioblastoma multiforme cancer cells is reported here for the first time. Furthermore, we performed COMPARE and hierarchical cluster analyses for 58 cell lines of the National Cancer Institute (NCI, United States) from nine different tumor types^1^. Then, we run bioinformatical gene promoter binding motif analyses for those genes obtained by COMPARE and hierarchical cluster analyses. Since AP-1 and NF-κB binding sites were predominant in the gene promoter sequences subjected to binding motif analyses, we used reporter cell lines for these two transcription factors and indeed observed that As_2_O_3_ inhibited promoter binding of these two transcription factors. Finally, we performed interactome network analysis using IPA software to identify cellular pathways that might be affected upon As_2_O_3_ treatment.

## Materials and Methods

### Cell Lines and Reagents

We tested cell lines expressing different modes of drug resistance and their corresponding parental, drug sensitive cell lines. These included the leukemia CCRF-CEM (drug sensitive), and the drug resistant CEM/ADR5000 (multidrug resistant), the human glioblastoma U87.MG (wild type) and U87.MG ΔEGFR (mutated type), and the colon carcinoma HCT-116 (p53^+/+^) and HCT-116 (p53^-/-^) cell lines. The details about the cell lines were described previously by our group (Saeed et al., 2014). Glioblastoma were provided from Dr. W. K. Cavenee (Ludwig Institute for Cancer Research, San Diego, CA, United States). While, colon cancer cell lines were provided by Dr. B. Vogelstein and H. Hermeking (Howard Hughes Medical Institute, Baltimore, MD, United States).

Tumor cell lines were routinely cultured in proper medium (Sigma–Aldrich, Taufkirchen, Germany) with 10% FBS (Sigma–Aldrich, Taufkirchen, Germany), 1% penicillin/streptomycin Sigma–Aldrich) (Bunz et al., 1998; Efferth et al., 2003). Moreover, doxorubicin (5000 ng/mL) was used to maintain the resistance of CEM/ADR5000 cells (Kimmig et al., 1990). While U87.MG ΔEGFR and HCT-116 (p53^-/-^) was treated with geneticin to maintain expression of the transcript. Cells were incubated at 37°C under 5% CO_2_ in a humidified atmosphere. As_2_O_3_ and geneticin were purchased from Sigma–Aldrich, Germany. Doxorubicin was kindly provided by the University Medical Center, Johannes Gutenberg University (Mainz, Germany).

### Cytotoxicity Assays

The cytotoxicity of As_2_O_3_ was assessed using the resazurin reduction assay method as previously described (Kuete et al., 2016, 2017). Cells were treated with different concentrations of As_2_O_3_, ranging from 0.003 to 500 μM. The assay principle is that resazurin (Sigma–Aldrich) is reduced by viable cells to the highly fluorescent resorufin (O’Brien et al., 2000). Dead cells have no capability to convert resazurin to resorufin which lack the fluorescence property. Infinite M2000 proplate reader (Tecan, Germany) detected the fluorescent signal that emitted by the viable cells. Experiments were done three times, with six replicates for each concentration. The viability of the cells treated with As_2_O_3_ was calculated as percentage in compared with untreated control cells. The obtained cell viability was plotted against As_2_O_3_ concentration using Microsoft Excel 2016. Dose response curve was used to calculate the IC_50_ values of every cell line.

### NF-κB Reporter Assay

HEK-Blue-Null1 cells were obtained from Invivogen (San Diego, CA, United States). It is derived from embryonic kidney-293 (HEK-293) cell line. HEK-Blue-Null1 cells culture conditions were previously described (Kadioglu et al., 2016b; Seo et al., 2016; Hamdoun and Efferth, 2017). Briefly, HEK-Blue-Null1 cells constantly transcribe the SEAP protein under control of the NF-κB promoter. Cells were treated with varying concentrations of As_2_O_3_. NF-κB pathway was activated using 100 ng/mL of TNF and inducted for 24 h. Then, detection using pre-warmed Quanti-Blue reagent (Invivogen) was performed according to the manufacturer’s protocol. SEAP was measured spectrophotometrically at 630 nm to detect NF-κB activity. The fold change was calculated in compare with untreated control cells. The NF-κB inhibitor triptolide (1 μM, Invivogen) was used as positive control. The experiments were repeated three times.

### AP-1 Reporter Assay

HEK293 cells were trypsinized and evenly distributed into 96 well plate polystyrene U96. The cells were then transfected with AP-1 luciferase reporter construct (CCS-011L, Qiagen, Germantown, MD, United States) using Attractene transfection reagent (Promega, Germany) according to the manufacturer’s protocols. After 24 h of the transfection process, cells were incubated with different doses of As_2_O_3_. After 18 h of incubation time with As_2_O_3_, AP-1 activation was induced by of 50 ng/ml phorbol 12-myristate 13-acetate PMA (Sigma–Aldrich) for 24 h. AP-1 promoter activity was measured by Dual-Luciferase Reporter Assay System (Promega, Madison, WI, United States) following the manufacturer’s recommendations. Both firefly and *Renilla* luciferase luminescences were measured using Infinite M2000 Pro plate reader (Tecan). The firefly luciferase luminescence ratio to *Renilla* luciferase luminescence for each sample was calculated to obtain the relative luciferase. Normalization of AP-1 activity was done using the following equation: relative luciferase of sample over the relative luciferase of the untreated control cells (Kadioglu et al., 2016a). AP-1 luciferase assay experiments were repeated twice.

### Bioinformatical Methods

In the present manuscript, we applied several methods of systems biology. While conventional medicine prefers a reductionist approach with one (or few) targets for an investigational drug, traditional medicine always emphasized the multi-specific nature of natural products (Efferth and Koch, 2011). The advent of genome-wide expression profiling techniques was estimated as specifically promising for natural product research, as complex cascades, pathways, and gross gene alteration patterns can be measured in a single experiment (Kadioglu and Efferth, 2014; Quan et al., 2014; Dos Santos et al., 2016; Fang et al., 2017). Molecular pharmacology with the investigation of single pathways has been enlarged by the new field of network pharmacology (Poornima et al., 2016; Schmidt and Efferth, 2016; Efferth et al., 2017). As myriads of data points are collected with genome-wide methods, the data evaluation requires the application of bioinformatics to uncover relevant biological mechanisms of drugs. The “-omics” technology in conjunction with bioinformatical methods allow the generation of hypothetical predictions that could be tested and verified in experimental and clinical settings.

#### COMPARE Analysis

A panel of 58 cell lines from National Cancer Institute (NCI), United States were used to perform COMPARE and hierarchical cluster analyses. Logarithmic IC_50_ values (log_10_IC_50_) of As_2_O_3_ have been deposited at the NCI database^2^. The mRNA expression values of NCI cell lines were determined via microarray analyses were deposited at the NCI website^2^ as well. These data were used to generate rank ordered lists of genes expressed in the NCI cell lines panel using COMPARE analyses (Paull et al., 1989).

To extract the most meaningful results from a non-relevant “background noise” of transcriptome-wide microarray-based mRNA hybridizations, we applied the COMPARE analysis, which has been developed by Paull et al. (1989) from the NCI (United States). During the past decades, NCI has investigated more than 300,000 compounds for their cytotoxic capability against a panel of 60 cell lines from different tumor origin. The still growing NCI drug repository does not contain only synthetic compounds but also natural products. Paull and his team observed that drugs with similar molecular modes of action reveal similar patterns of growth inhibition based on their log_10_IC_50_. This correlation was used to develop an automated algorithm based on the Pearson correlation rank test. The details of the COMPARE methodology have been described (Paull et al., 1989, 1995; Zaharevitz et al., 2002). The use of the COMPARE algorithm to analyze data from the NCI human tumor cell line screen emerged more and more as a standard tool in drug discovery.

COMPARE analysis can be applied to identify inhibitors for a given mechanism of interest (e.g., inhibitors of EGFR, tubulins, DNA topoisomerases and others) (Wosikowski et al., 1997; Pfister et al., 2009). Vice versa, COMPARE analyses can also be used to suggest possible modes of action and/or determinants of resistance for compounds of interest (e.g., *P*-glycoprotein, p53 or Ras) (Lee et al., 1994; Alvarez et al., 1995; Koo et al., 1996; O’Connor et al., 1997).

COMPARE analyses were performed with software implemented into the web site of the NCI^3^. COMPARE analyses yielded rank-ordered lists of compounds. To obtain COMPARE rankings, a scale index of similarity between log_10_IC_50_ values of As_2_O_3_ and the transcriptome-based mRNA-based gene for the NCI panel of cell lines has been generated. The mRNA microarray hybridization of the NCI cell lines has been reported and deposited at the NCI Web site^4^ (Scherf et al., 2000; Amundson et al., 2008).

COMPARE analyses were performed to produce rank-ordered lists of genes expressed in the NCI cell lines. The results are sorted by the correlation coefficient (*R*-values). The methodology has been described previously in detail as a tool to identify candidate genes for drug resistance and sensitivity (Evans et al., 2008; Fagan et al., 2012; Luzina and Popov, 2012). Greater mRNA expression correlated with enhanced drug resistance in the standard COMPARE approach, whereas greater mRNA expression in cell lines indicated drug sensitivity in reverse COMPARE analyses. Pearson’s correlation test was used to calculate significance values and rank correlation coefficients as a relative measure for the linear dependency of two variables.

#### Hierarchical Cluster Analyses

Another wide used methodology to extract relevant results from transcriptomic data sets are cluster analyses, which represent distance/proximity-based approaches to unravel structures in large data sets. In systems biology, cluster analyses are valuable to define profiles of genes (“gene signatures”), whose expression is linked to biological phenomena, i.e., histological subtypes of tumors, resistance or sensitivity of tumors toward anticancer treatments, survival chances of cancer patients etc. Among the numerous clustering methods (e.g., topological interaction models, influence maps, physical regulatory maps, self-organizing maps, principal component analysis etc.), supervised, hierarchical and aggregative techniques provide advantages for pharmacological questions in cancer biology and pharmacology, because of their flexibility, possibility to include biological knowledge with different weighting, and detection of higher-order relationships between clusters of profiles (Mocellin et al., 2005). Aggregative hierarchical clustering represents a frequently method to investigate gene expression signatures (Eisen et al., 1998; Perou et al., 2000; Ross et al., 2000).

In the present study, we performed hierarchical cluster Analysis to group heterogeneous objects into clusters of homogeneous objects. All objects are assembled into a cluster tree (dendrogram). Thus, objects with tightly related features appear together, whereas the separation in the cluster tree increases with progressive dissimilarity. The merging of objects with similar features leads to formation of a cluster, the shortest the distance of the branch the closest degree of relatedness (Kadioglu and Efferth, 2015). Hierarchical clustering and heat map analysis were conducted using clustered image map (CIM) miner software. One matrix CIM^4^ and Ward method were performed. Importantly, COMPARE analyses and cluster models have been validated for gene expression profiling, identification of candidate genes for drug resistance and sensitivity and for understanding molecular biology of cancer (Efferth et al., 1997; Scherf et al., 2000).

#### Interactome Network Analysis

The biomedical literature has been flooded with genomic and transcriptomic data, which urgently need to be organized in bioregulatory networks comparable to electronic circuit programs. The rationale to generate regulatory network models is that pathophysiological processes may be associated with unexpected traits that are not amenable to standard statistical techniques. Building reliable regulatory networks from large-scale genomic and transcriptomic will considerably improve the identification of novel signaling routes and therapeutic targets, since both may differ in healthy and diseased cells (Lefebvre et al., 2012). Hence network approaches will be indispensable for patient-tailored precision medicine to predict drug response and overcome drug resistance (Ganter and Giroux, 2008).

Therefore, a number of software programs have been developed to address this problem, including programs developed at academia such as network molecular interaction maps (MIMs) (Kohn, 1999, 2001; Kohn et al., 2006a,b; Luna et al., 2011), Cytoscape, (Ideker et al., 2001), CellDesigner (Funahashi et al., 2003), TranscriptomeBrowser (Lopez et al., 2008; Lepoivre et al., 2012), PathVisio-Validator (Chandan et al., 2012), Systems Biology Graphical Notation (Ma et al., 2014) as well as commercial products, e.g., Biocarta, Ingenuity Pathway analysis, Pathway Studio, MetaCore and many others. All these programs reveal advantages and disadvantages (Thomas and Bonchev, 2010; Wang et al., 2013).

In the present investigation, genes described in the literature as factors determining cellular responsiveness to As_2_O_3_ (**Table 5**) plus the genes identified via compare analysis of our present and previous investigations on As_2_O_3_ (Efferth and Kaina, 2004) were chosen to perform network analyses using Ingenuity Pathway Analysis (IPA) (Qiagen Bioinformatics, Redwood City, CA, United States), as IPA is one of the most used programs to generate bioregulatory networks. We performed network analysis using two different approaches: manually curated pathways and software-based analyses. Published information from literature was used to generate the network and to cluster the genes. IPA Pathway Designer was used to construct the network and the pathways where our genes contributed to.

#### Gene Promoter Transcription Factor Motif Analysis

Forty genes from the COMPARE analyses results significantly associated with log_10_IC_50_ values of As_2_O_3_ for the 58 cell lines. UCSC Genome Browser Gene^5^ was used to analyze the 40 genes to obtain gene’s promoter sequences up to 50 kilobases upstream of the transcription start site. The results were saved on BED (Browser Extensible Data) files for further use (Seo et al., 2016).

The bed file was uploaded on Galaxy, Cistrome software (Liu et al., 2011) to detect the possible transcription factor binding motifs for the selected genes. The software is available at http://cistrome.dfci.harvard.edu/ap/root. Integrative analysis using SeqPos tool was applied to screen the motif binding site on the uploaded genes promoter sequences. Both, UCSC Genome Browser and Galaxy, Cistrome are free softwares. SeqPos tool scans all the motifs that are available on the following databases Transfac, JASPAR, UniPROBE (pbm), hPDI database. The result of the screening was ordered by -log_10_ (*P*-value).

### Statistical Analysis

Pearson’s correlation test was applied to calculate significance correlation in COMPARE and hierarchical cluster analyses. The chi-squared test was performed to investigate the linear dependency between the resistance and the sensitivity of the cell lines panel. Student’s *t*-test (Microsoft Excel 2016) was applied to determine the statistical significance of As_2_O_3_ effect on AP-1 and NF-κB transcription factors. A level of *P* < 0.05 was revealed as statistically significant. All data were shown as mean values ± SD.

## Results

### Cytotoxicity

**FIGURE 1:**
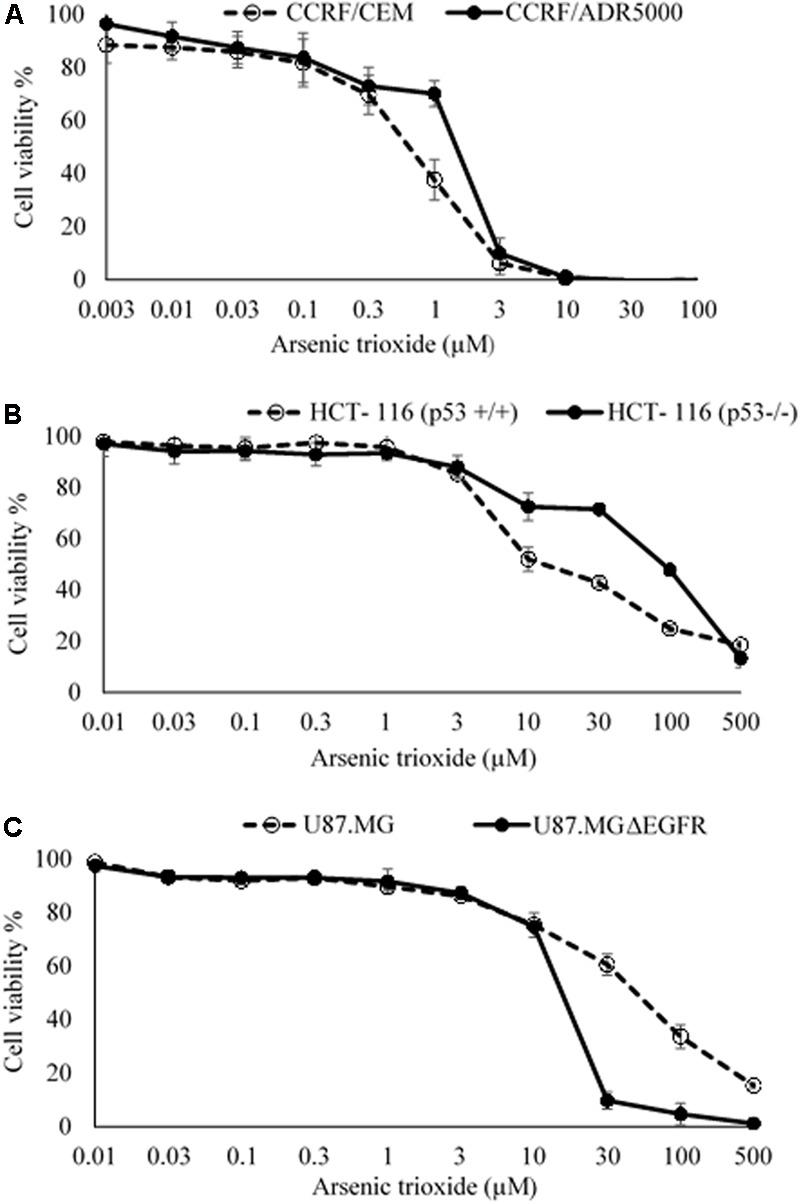
Growth inhibition of sensitive and drug-resistance cancer cell lines treated with different concentrations of As_2_O_3_. **(A)**. CCRF-CEM and *P*-glycoprotein-expressing CEM/ADR5000 leukemia cell lines; **(B)** HCT-116 (p53^+/+^) wild-type and HCT-116 (p53^-/-^) knockout colon carcinoma cell lines; **(C)** U87.MG wild-type and EGFR-transfected U87.MG ΔEGFR glioblastoma cell lines. The dose response curves show mean values ± SD of three independent experiments with each six parallel measurements.

The cytotoxic effect of As_2_O_3_ was determined using resazurin reduction assay. As_2_O_3_ was applied on three different resistance mechanisms (*P*-glycoprotein overexpression, mutant EGFR, knockout p53). The IC_50_ values for CCRF-CEM cells and CEM/ADR5000 cells were 0.48 ± 0.22 μM and 0.90 ± 0.38 μM, respectively. For CEM/ADR5000 the degrees of resistance was 1.88. Additionally, As_2_O_3_ was tested against HCT116 p53 knockout cells (p53^-/-^) and their wild-type cells, HCT116 (p53^+/+^). The HCT116 (p53^-/-^) cells exhibited resistance toward As_2_O_3_ with an IC_50_ value of 90.83 ± 0.83 μM, which was higher than the IC_50_ value of wild-type HCT116 (p53^+/+^) cells (12.68 ± 0.78 μM). Interestingly, glioblastoma cell line (U87.MG) were even more sensitive to As_2_O_3_ than U87.MG wild-type cells with IC_50_ values of 15.60 ± 0.93 and 47.82 ± 1.25 μM, respectively. The degree of resistance was 0.33. The Dose response curves and IC_50_ values for the above-mentioned cell lines are shown in **Figure 1** and **Table 1**.

**Table 1:** IC_50_ values of As_2_O_3_ toward various tumor cell lines.

Cell lines	As_2_O_3_	
	IC_50_ (μM)	Degree of resistance
CCRF-CEM	0.48 ± 0.22	1.88
CEM/ADR5000	0.90 ± 0.38	
U87.MG	47.82 ± 1.25	0.33
U87.MG ΔEGFR	15.60 ± 0.93	
HCT116 (p53^+/+^)	12.68 ± 0.74	7.16
HCT116 (p53^-/-^)	90.83 ± 0.83	

*The data is shown as mean values ± SD of three independent experiments with each six parallel measurements. Degrees of resistance were calculated by dividing the IC_50_ value of resistant cell line over the IC_50_ value of sensitive cell line.*

### COMPARE and Hierarchical Cluster Analyses

**FIGURE 2:**
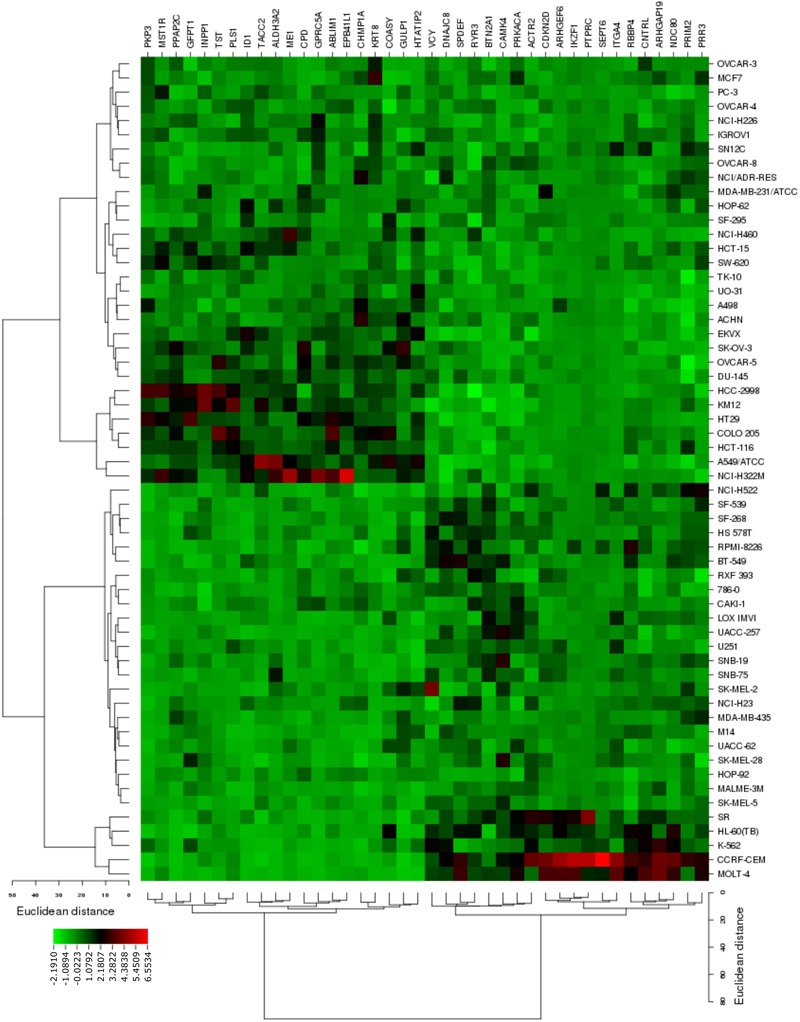
Heat map obtained by cluster analysis from microarray-based mRNA expression profiles of genes correlating with cellular responsiveness to As_2_O_3_. The analysis shows the clustering of 58 NCI tumor cell lines.

The transcriptome-wide mRNA expression of the 58 NCI cell lines were subjected to COMPARE analyses based on Pearson’s rank correlation tests. In this test, the microarray expression data were correlated with the log_10_IC_50_ values of As_2_O_3_ for the 58 cell lines. This bioinformatics approach permitted the identification of molecular factors that liked to cellular response to As_2_O_3_. The top 20 genes with positive correlation coefficients and top 20 genes with negative correlation coefficients are shown in **Table 2**. These 40 genes were used to conduct hierarchical cluster analysis (cluster image mapping) to predict, whether these genes may play a role in sensitivity or resistance of the cells to As_2_O_3_. The resulting dendrogram with the 58 cell lines applied cluster analyses depicted on the left of the heat-map, while distribution of the 40 genes are shown on the top of the heat-map (**Figure 2**). The dendrogram can be divided into four major clusters for As_2_O_3_. Based on chi-square test we looked whether the clustering of cell was associated with cellular responsiveness of the cell lines to As_2_O_3_. Indeed, the branching of sensitive or resistant cell lines was statistically significant (**Table 3**). The genes identified via this approach belong to diverse groups such as cell cycle progression and proliferation genes including (*PRKACA*, ARHGAP19, *CDKN2D* etc.), tumor suppressor related genes (*RBBP4, CHMP1A*, and *GPRC5A*), cytoskeleton related genes (*PTPRC, ABLIM1, EPB41L1* and *PLS1*), metabolic pathway genes (*ME1, COASY*, and *ALDH3A2*), signal transduction genes (*PPAP2C* and *PKP3*) and apoptosis-regulating gene (ID1).

**Table 2:** Correlation coefficients of mRNA expression to log_10_ IC_50_ values identified by COMPARE analyses for 58 NCI tumor cell lines and functions of the identified genes.

Coefficient	Symbol	Name	Function
0.568	*PRKACA*	Protein kinase, cAMP-dependent, catalytic, α	Affects cell cycle progression and proliferation by downregulation of CDKN1B.
0.507	ARHGAP19	Rho GTPase activating protein 19	Involved in cell migration, proliferation, actin remodeling, differentiation, and G1 cell cycle progression.
0.487	*CAMK4*	Calcium/calmodulin-dependent protein kinase IV	Role in spermatogenesis by phosphorylating protamines.
0.485	*BTN2A1*	Butyrophilin, subfamily 2, member A1	Involved in cell proliferation and development.
0.483	*SEPT6*	Septin 6	Involved in cytokinesis
0.475	*ITGA4*	Integrin, α 4 (antigen CD49D, α 4 subunit of VLA-4 receptor)	Cell surface adhesion receptor mediating cell-adhesion to extracellular matrix or to other cells.
0.475	*CNTRL*	Centrosomal protein 110 kDa	Involved in cell cycle progression and cytokinesis.
0.471	*DNAJC8*	Platelet-activating factor receptor	Unknown
0.47	*SPDEF*	SAM pointed domain containing ets transcription factor	Tumor metastasis suppressor.
0.47	*RYR3*	Ryanodine receptor 3	Calcium release from intracellular storage.
0.469	*IKZF1*	IKAROS family zinc finger 1 (Ikaros)	Implicated in the control of lymphoid development.
0.467	*ACTR2*	ARP2 actin-related protein 2 homolog (yeast)	Role for cell shape and motility.
0.463	*PTPRC*	Protein tyrosine phosphatase, receptor type	Structural constituents of cytoskeleton, involved in cell organization/biogenesis.
0.459	*PRIM2*	Primase, DNA, polypeptide 2 (58 kDa)	DNA primase subunit, key enzyme in DNA replication.
0.457	*RBBP4*	Retinoblastoma binding protein	Inhibitor of TGFB1 activity, plays a role in oncogenesis.
0.454	*PRR3*	Proline rich 3	Nucleic acid binding activity.
0.452	*CDKN2D*	Cyclin-dependent kinase inhibitor 2D (p19)	Cell growth regulator controlling G1 cell cycle progression.
0.452	*ARHGEF6*	Rac/Cdc42 guanine nucleotide exchange factor (GEF) 6	Activator of Ras-like family of Rho proteins by exchanging bound GDP for GTP.
0.45	*NDC80*	NDC80 homolog, kinetochore complex component (*S. cerevisiae*)	Component of the essential kinetochore-associated NDC80 complex, which is required for chromosome segregation and spindle checkpoint activity.
0.448	VCY	Variable charge, Y-linked	May mediate spermatogenesis processes or may contribute to sex ratio distortion.
0.435	*IRF2*	Interferon regulatory factor 2	Repressor of IRF1, transcriptional activator of histone H4, regulates NFκB activity.
-0.71	*PPAP2C*	Phosphatidic acid phosphatase type 2C	Converts phosphatidic acid to diacylglycerol, function in *de novo* synthesis of glycerolipids phospholipase D-mediated signal transduction.
-0.566	*ALDH3A2*	Aldehyde dehydrogenase 3 family, member A2	Catalyzes the oxidation of long-chain aliphatic aldehydes to fatty acids.
-0.542	*ME1*	Malic enzyme 1, NADP^+^-dependent, cytosolic	Mitochondrial NAD-dependent malic enzyme, catalyzing the oxidative decarboxylation of malate to pyruvate.
-0.539	*COASY*	CoA synthase	Coenzyme A (CoA) is a carrier of acetyl and acyl groups in cells and is crucial for numerous metabolic pathways.
-0.533	*PKP3*	Plakophilin 3	Mediating protein-protein interactions. Playing a role in junctional plaques.
-0.531	*ID1*	Inhibitor of DNA binding 1, dominant negative helix-loop-helix protein	Implicated in regulating a variety of cellular processes, including cellular growth, senescence, differentiation, apoptosis, angiogenesis, and neoplastic transformation.
-0.517	*GPRC5A*	G protein-coupled receptor, family C, group 5, member A	Functions as a tumor suppressor in lung cancer. May play a role in the proliferation of breast cancer cells.
-0.513	*TST*	Thiosulfate sulfurtransferase (rhodanese)	May contribute to cyanide detoxification.
-0.496	*ABLIM1*	Actin binding LIM protein 1	Putatively bridging the actin-based cytoskeleton with LIM protein-binding partners.
-0.493	*CPD*	Carboxypeptidase D	Carboxypeptidase, involved in secretory pathways.
-0.487	*CHMP1A*	Chromatin modifying protein 1A	Role in nuclear gene silencing, tumor suppressor, regulates tumor growth by the TP53 signaling pathway.
-0.486	*GFPT1*	Glutamine–fructose-6-phosphate transaminase 1	Involved in regulating the availability of precursors for *N* and *O*-linked glycosylation of proteins.
0.483	*EPB41L1*	Erythrocyte membrane protein band 4.1-like 1	Critical for the membrane-associated cytoskeleton structure of erythrocytes.
-0.48	*TACC2*	Transforming, acidic coiled-coil containing protein 2	Playing a role in organizing centrosomal microtubules, tumor progression marker.
-0.479	*INPP1*	Inositol polyphosphate-1-phosphatase	Involved in response to lithium prophylaxis and treatment of bipolar disorders.
-0.477	*MST1R*	Macrophage stimulating 1 receptor (c-met-related tyrosine kinase)	Involved in development of epithelial tissue as well as cell proliferation, cell survival, and cell motility in both normal and disease states.
-0.476	*PLS1*	Plastin 1	Actin-bundling protein in the absence of calcium.
-0.475	*KRT8*	Keratin 8	Role in maintaining cellular structural integrity.
0.475	*GULP1*	GULP, engulfment adaptor PTB domain containing 1	Adaptor protein required for efficient engulfment of apoptotic cells by phagocytes.

**Table 3:** Separation of clusters of NCI cell lines obtained by hierarchical cluster analyses for As_2_O_3._

	Sensitive	Resistant
AS_2_O_3_		
Partition (log_10_IC_50_)	<-5.45 M	>-5.45 M
Cluster 1	6	17
Cluster 2	0	7
Cluster 3	18	5
Cluster 4	5	0
χ^2^ Test	*P* = 1.86 × 10^-5^	

*The median log_*10*_IC_*50*_ value (*M*) for As_*2*_O_*3*_ was used as a cut-off to separate cancer cell lines as being “sensitive” or “resistant.”*

### Gene Promoter Analysis for Transcription Factor Binding Motifs

Using the computational method of Galaxy Cistrome analysis, we investigated DNA promoter sequences of deregulated genes found in NCI microarray data. Forty genes were uploaded to Galaxy Cistrome for SeqPos motif scan. These genes are contribute in cell cycle progression and proliferation, cytokinesis, metabolic pathways, cell shape, apoptosis and signal transduction. The top 20 pronounced motifs are illustrated in **Table 4**. Interestingly, AP-1 and NF-κB were among the top transcription factors that possibly bind to the promoters with *z*-scores of -5.456 and -5.268, respectively. Both factors play critical role in the cancer therapy.

**Table 4:** Top 20 transcription factor promoter binding motifs for genes identified by COMPARE analysis (see **Table 3**).

Clusters	Factor	*z*-score	-10^∗^log(*p*-value)
1	YY1	**-**7.388	302.304
	Zfp42	**-**6.184	218.837
2	PIF3	**-**6.798	259.645
	Tye7	**-**3.945	101.291
3	JUND	**-**6.447	235.883
	**AP-1**	**-**5.456	175.291
	AP-1	**-**5.034	152.444
	AP-1	**-**4.834	142.187
	AP-1	**-**4.286	116.074
	GCN4	**-**4.208	112.612
	BACH2	**-**3.219	73.482
4	Hic1	**-**5.884	200.294
5	EBF1	**-**5.841	197.699
6	MYB	**-**5.793	194.848
	Myb	**-**3.468	82.468
7	REB1	**-**5.788	194.519
8	STAT1	**-**5.636	185.601
9	Ets1	**-**5.585	182.653
10	Ets1	**-**4.274	115.545
	Ets1	**-**4.047	105.592
	ELK4	**-**3.344	77.908
	Etv5	**-**3.298	76.284
	Etv4	**-**3.135	70.602
	TAF-1	**-**5.508	178.251
11	HBP-1a	**-**4.112	108.405
	TAF-1	**-**3.659	89.741
	CPRF-1	**-**3.21	73.169
	LTF	**-**5.483	176.81
12	NEUROD1	**-**5.445	174.695
13	MYF	**-**3.482	82.983
	Myf	**-**3.482	82.983
	LMO2	**-**5.319	167.711
14	Tcfe2a	**-**5.29	166.101
	AREB6| ZEB1	**-**4.582	129.833
	ATOH1	**-**3.199	72.778
	**NF-κB**	**-**5.268	164.89
15	**NF-κB**	**-**4.056	105.966
	P50:P50	**-**3.365	78.671
	MZF1_1-4	**-**5.19	160.702
16	Churchill	**-**3.25	74.581
	UBP1	**-**5.046	153.04
17	S8| Prrx2	**-**5.034	152.432
18	Msx-2	**-**4.391	120.844
	Gbx2	**-**3.887	98.882
	Msx2	**-**3.79	94.95
	HOXD1	**-**3.298	76.274
	Barx-2	**-**3.192	72.547
	Prrx2	**-**3.191	72.514
	SP3	**-**5.015	151.433
19	TIMELESS	**-**5.014	151.355
20	Egr| Egr1	**-**4.994	150.332

*The binding motifs have been identified using Galaxy Cistrome.*

### AP-1 Reporter Assay

**FIGURE 3:**
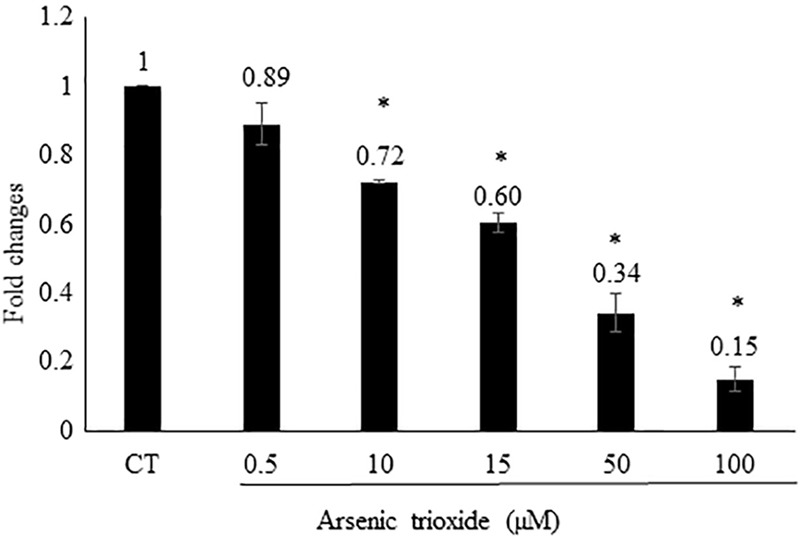
Effect of As_2_O_3_ on transfected HEK-293 cells with an AP1 luciferase reporter construct. The AP1 reporter cell line was treated with five different concentrations of As_2_O_3_. The quantification was done after 24 h incubation in comparison to untreated control cells (CT). Mean value standard deviation of triplicate measurements are shown. ^∗^Indicates statistical significant in compare to control cells at level of *P* < 0.05.

Based on this finding, we assume that As_2_O_3_ may affect AP-1. To verify this hypothesis, we generated an AP-1 reporter cell line. The transfected HEK293 was incubated with five different concentrations of As_2_O_3_ to find out, whether AP-1 promoter activity was inhibited. Treatment of transfected cells with As_2_O_3_ significantly inhibited the PMA-mediated AP-1 activity in a dose-dependent manner as shown in **Figure 3**.

### NF-κB Reporter Assay

**FIGURE 4:**
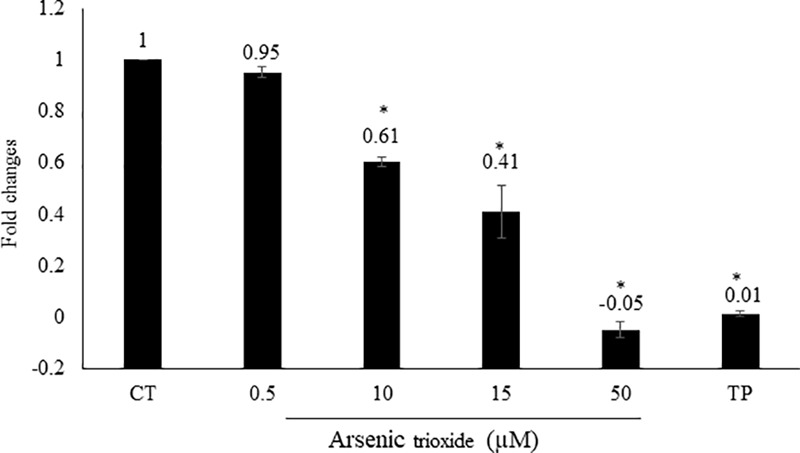
Effect of As_2_O_3_ on NF-κB activity. The HEK-Blue^TM^ cell line was treated with four different concentrations of As_2_O_3_ or 1 μM triptolide (TP) as positive control. The quantification was done after 1 h incubation in comparison to untreated control cells (CT). Mean value standard deviation of triplicate measurements are shown. ^∗^Indicates statistical significant in compare to control cells at level of *P* < 0.05.

Interestingly, the capability of As_2_O_3_ to affect NF-κB is debatable point in the literature. As_2_O_3_ can act as activator or inhibitor NF-κB (Woo et al., 2004; Han et al., 2005). From our motif analysis, we obtained NF-κB among the top 20 transcription factors. Therefore, we investigated whether As_2_O_3_ is able to inhibit NF-κB signaling. To achieve this, we performed a SEAP-driven NF-κB reporter cell line. Indeed, As_2_O_3_ inhibit NF-κB in a comparable manner as the NF-κB inhibitor, triptolide. The fold change was statistically significant for three As_2_O_3_ concentrations (10, 15, and 50 μM) as demonstrated in **Figure 4**.

### Interactome Network Analysis

**FIGURE 5:**
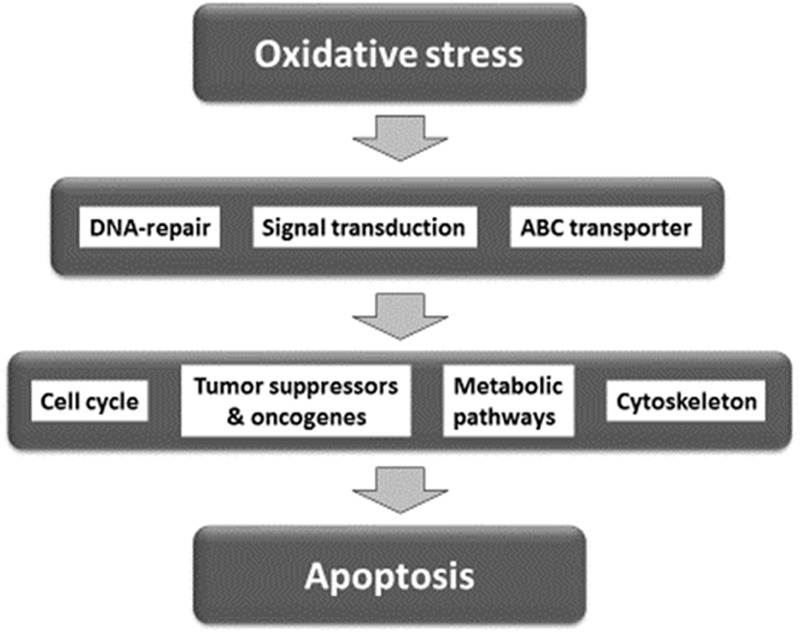
Synopsis of mechanisms involved in the activity of As_2_O_3_ against cancer cells.

For network analysis, we subjected the transcriptomic data of our present and previous investigations on As_2_O_3_ (Efferth and Kaina, 2004) as well as of factors determining cellular responsiveness to As_2_O_3_, which are known from the literature. In addition to the well-known target of As_2_O_3_ in APL, the PML-RARAα fusion protein (Goto et al., 2011; Tomita et al., 2013; Chendamarai et al., 2015; Lou et al., 2015; Liu J. et al., 2016), numerous other determinants of resistance have been described in the literature (**Table 5**). Firstly, we performed a manually curated analysis of the genes by visual inspection of the genes and assigning then to their specific functions. The main functional groups were oxidative stress response, DNA repair, regulation of cell cycle/proliferation, signal transduction, drug transporter, cytoskeletal elements, tumor suppressors/oncogenes, metabolic pathways, and apoptosis. Based on this analysis, a presumable integrative interaction network was constructed (**Figure 5**).

**Table 5:** Compilation of determinants of cellular responsiveness of cancer cells toward As_2_O_3_.

Genes/Proteins	Reference
**Oxidative stress response:**	
	Present investigation;
GSH	Yang et al., 1999; Davison et al., 2003; Hour et al., 2004; Lee et al., 2006; Han et al., 2008; Matulis et al., 2009; Matulis et al., 2012
*SOD (Cu/Zn)*	Hour et al., 2004
*NXN, TXNRD1*	Efferth and Kaina, 2004
*BACH2*	Zhou et al., 2005
γ-glutamyltransferase	Giommarelli et al., 2009
HO-1	Zhou et al., 2005
HIF-1α	Tung et al., 2011; Liu H. et al., 2016
GST-π	Zhao et al., 2011
**Drug transport:**	
*ABCC1 (MRP1);ABCC2 (MRP2); ABCC4 (MRP4*)	Seo et al., 2007; Chen et al., 2009; Yuan et al., 2016
*ASAN1*	Chen et al., 2009
**DNA repair:**	
*SMC2L1*	Efferth and Kaina, 2004
hMSH2	Hour et al., 2004
**Cell cycle/proliferation:**	
*ARHGAPAP19, CDKN2D, PRKACA*,	Present investigation
*TFDP2, ZNF151*	Efferth and Kaina, 2004
*p21WAF/CIP*	Hour et al., 2004
*BTBD2, IGFBP1*	Zhou et al., 2005
*CCND1*	Roszak et al., 2013
AURKB	Yih et al., 2013
**Tumor suppressors/oncogenes:**	
*RBBP4, CHMP1A, GPRC5A*	Present investigation
PML/RARAα	Goto et al., 2011; Tomita et al., 2013; Chendamarai et al., 2015; Lou et al., 2015; Liu J. et al., 2016
p53	Yan et al., 2011; Zhang et al., 2013; Zheng et al., 2014
EGFR	Zhang et al., 2012
*MYC*	Hour et al., 2004
**Signal transduction:**	
*CAMK4, PPAP2C, PKB3*	Present investigation
AP-1 (Fos/Jun)	Present investigation
*DDEF2, SDC1, SH2BP3, STMN1, TJP1*	Efferth and Kaina, 2004
PI3K, PKC, AKT	Tabellini et al., 2005; Roszak et al., 2013; Yih et al., 2013; Amigo-Jimenez et al., 2015
NFKB1	Efferth and Kaina, 2004
**Metabolic pathways:**	
*ME1, COAS4, ALDH3A2*	Present investigation
*ALDH3A2*	Efferth and Kaina, 2004
*ALDH3A1*	Zhang et al., 2015
**Cytoskeleton:**	
*PTPRC, ABLIM1, EPB41L1, PLS1*	Present investigation
*ACAA2, ARHGEF7, KRT8, MYL3*	Efferth and Kaina, 2004
**Apoptosis:**	
*ID1*	Present investigation
*GRB7, PIGPC1*	Efferth and Kaina, 2004
Bcl2, Noxa	Hour et al., 2004; Matulis et al., 2012
Mcl-1	Amigo-Jimenez et al., 2015

**FIGURE 6:**
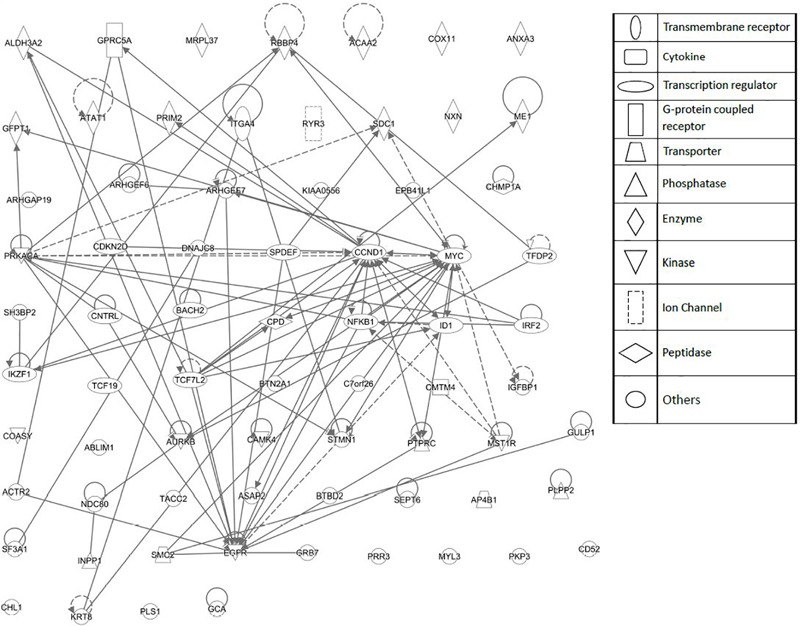
Network pharmacology for genes determining cellular responsiveness to As_2_O_3_ using Ingenuity Pathway Analysis. Dashed and solid lines indicates indirect and direct interactions, respectively.

**FIGURE 7:**
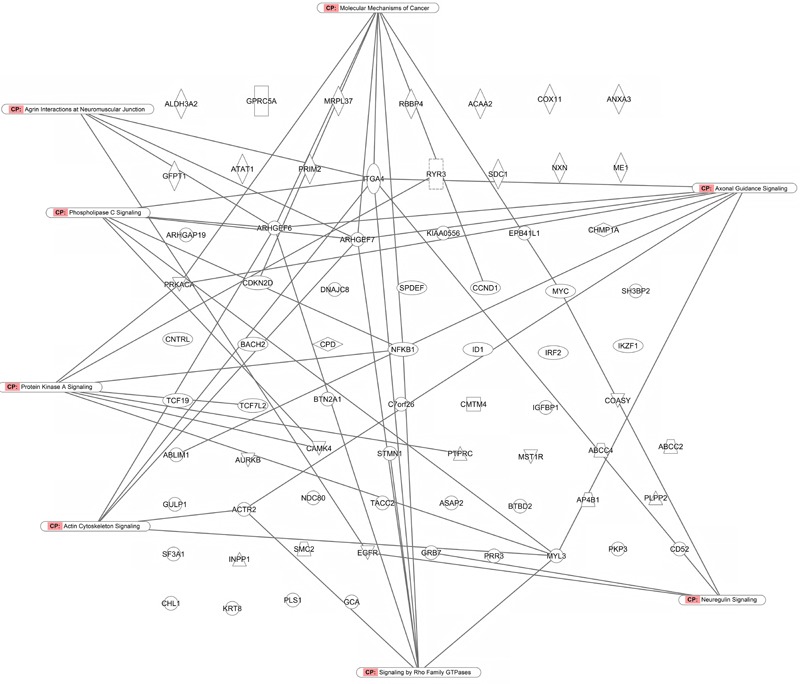
Canonical pathway analysis for genes determining cellular responsiveness to As_2_O_3_ using Ingenuity Pathway Analysis.

**FIGURE 8:**
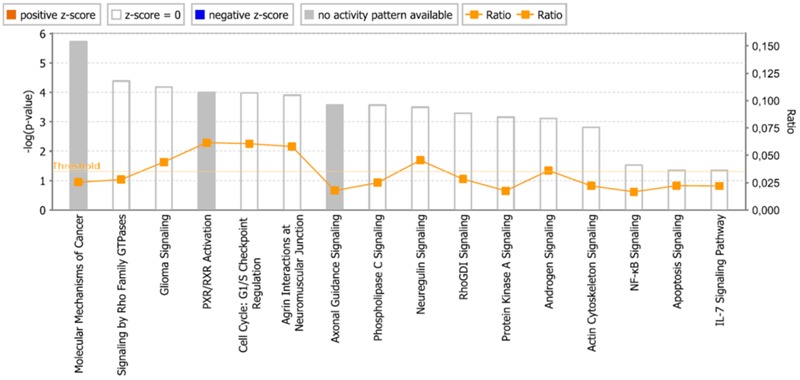
Pathways identified by IPA. Significance *P*-values were calculated based on the Fisher’s right tailed exact test. The –log (*p*-value) are shown on the *y*-axis of the bar chart. The color of the bars indicates the activity (orange bars) or the inhibition (blue bars) of the predicted pathways. In this analysis only significant results was shown. By default IPA applies a –log (*p*-value) cutoff of 1.3 (threshold). Pathways with a *p*-value equal to or greater than (less significant than) 0.05 are not shown. The orange and blue colored bars indicate predicted pathway activation, or predicted inhibition, respectively (*z*-score). White bars are those with a *z*-score at or very close to 0. Gray bars indicate pathways, where no prediction can currently be made.

As a second step, we subjected the assembled genes to Ingenuity Pathway Analysis. The interactome map obtained was rather complex (**Figure 6**). Therefore, we refined the analysis by analyzing the canonical pathways. The identified pathways belonged to signal transduction (phospholipase C signaling, protein kinase A signaling, signaling by Rho family GTPases, actin cytoskeleton signaling), molecular mechanisms of cancer, neurological pathways (axonal guidance signaling, neuroregulin signaling, agrin interactions with neuromuscular junctions), PXR/RXR activation, IL-7 signaling pathway and androgen signaling (**Figure 7**). The significance of this pathway are presented as –log (*p*-value) in **Figure 8**.

**FIGURE 9:**
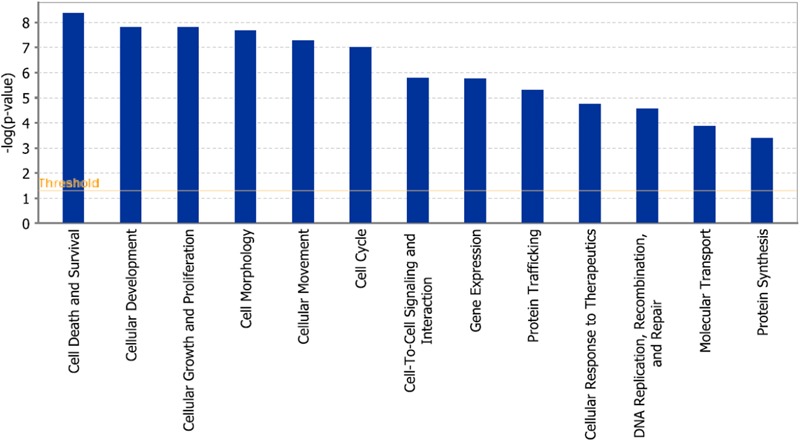
Statistically significant biological function analysis using IPA.

**FIGURE 10:**
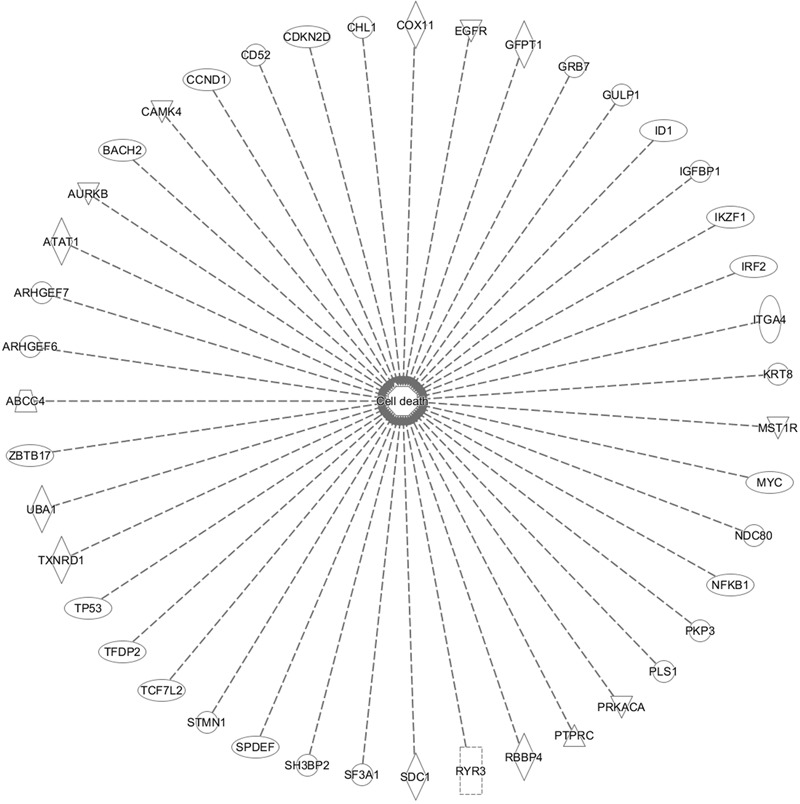
Set of 43 genes that involve in cell death function. This gene is duplicated on the inserted dataset.

In addition, cell death and survival, cell cycle, cell morphology, cellular movement, cellular development were identified by IPA as biological functions from our selected genes (**Figure 9**). A network of 36 selected genes that involve in the cell death (apoptosis) as the top biological function is shown in **Figure 10**.

While the importance of signaling processes was obvious by both visually and IPA-guided analyses, both approaches revealed additional functional groups complementing each other and highlighting the complexity of mechanisms contributing to the cytotoxicity of As_2_O_3_.

## Discussion

In the current study, we investigated the potential of As_2_O_3_ to treat drug-resistant tumor cells. Tumors frequently develop resistance to a broad spectrum of anti-cancer drugs (Efferth et al., 2008). Therefore, drug resistance represents an important obstacle to defeat cancer. In this study, we showed that As_2_O_3_ revealed profound cytotoxicity toward various cancer cell lines.

Our results showed that multidrug-resistant CEM/ADR5000 cells which overexpress *P*-glycoprotein did not reveal cross-resistance to As_2_O_3_. Therefore, *P*-glycoprotein did not play an important role for resistance cellular responsiveness to As_2_O_3_. Our results are in accordance with those of (Hu et al., 2003), who also did not find cross-resistance of multidrug-resistant *P*-glycoprotein-expressing cell lines toward As_2_O_3_. This finding is also corroborated by data generated by Sertel et al. (2012). As_2_O_3_ counteracted multidrug resistance by interference with drug resistance genes. The expression of DNA topoisomerase 2 was increased and of glutathione S-transferase-π and Bcl-2 decreased upon treatment with As_2_O_3_ (Zhao et al., 2011)_._ Other ATP-binding cassette (ABC) transporters than *P*-glycoprotein might be more important for As_2_O_3_. Treatment with As_2_O_3_ lead to the induction of ABCC1, but not *P*-glycoprotein (Seo et al., 2007) and As_2_O_3_-resistant cell lines overexpressed *P*-glycoprotein but also ABCC1, ABCC2, and ABCC4 (Chen et al., 2009, 2016; Yuan et al., 2016). ABCC1-overexpressing tumor cells accumulated less As_2_O_3_ than wild-type cells (Salerno and Garnier-Suillerot, 2003).

To the best of our knowledge, the anti-proliferative activities of As_2_O_3_ and particularly its effect against p53 knockout HCT116 (p53^-/-^) colon carcinoma and EGRF mutant U87.MG ΔEGFR glioblastoma multiforme cell lines are being tested for the first time.

TP53 maintains cellular integrity by regulating cell cycle arrest, DNA repair and apoptosis. Functional loss by TP53 mutations lead not only to carcinogenesis, but to drug and radio resistance too (Hientz et al., 2017). In our investigation, HCT116 (p53^-/-^) knockout cells exhibited resistance toward As_2_O_3_. Our data are in agreement with other investigations with isogenic cell lines (Yan et al., 2011; Zhang et al., 2013), but not with cell lines with different p53 status and different genomic background (Liu et al., 2003; Zhao et al., 2008). This indicates that the p53 mutational status in correlation studies with different cell lines may be overruled by other mechanisms of resistance to As_2_O_3_. Therefore, the targeted loss of p53 function by knockout was more suitable to undoubtedly elucidate the role of p53 as resistance factor for As_2_O_3_. A role of p53 mutations can also be deduced from cells resistant to As_2_O_3_, which acquired p53 mutations and which were re-sensitized to As_2_O_3_ by a small molecule inhibitor specifically targeting mutant p53, nutlin-3 (Zheng et al., 2014).

Furthermore, we found that U87.MG cells showed collateral sensitivity to As_2_O_3_. EGFR plays a significant role in cancer progression, not only by transducing growth signals into cancer cells, but also by triggering invasion and metastasis (Baselga, 2002; Lui and Grandis, 2002). Inhibition of EGFR leads to the induction of apoptosis. This may explain, how As_2_O_3_ suppresses EGFR-expressing tumor cells (Efferth and Kaina, 2004). The phenomenon of collateral sensitivity (hypersensitivity) offers an attractive strategy to overcome drug resistance (Pluchino et al., 2012). Moreover, collateral sensitivity may open the chance for combination therapy of known anticancer drugs with As_2_O_3_ to efficiently kill drug- resistant tumors with EGFR over expression. The reason for the collateral sensitivity is not yet known, but a direct interaction of As_2_O_3_ with EGFR could be suggested, since it has been reported that As_2_O_3_ down-regulated EGFR expression in a dose-dependent manner (Zhang et al., 2012).

We further evaluated the molecular factors of sensitivity and resistance of cancer cell lines toward As_2_O_3_. We correlated the log_10_IC_50_-values with the microarray- based mRNA expression profiles of 58 tumor cell lines by COMPARE analysis. Genes from diverse functional groups have been identified. Previously, the effect of As_2_O_3_ on mRNA expression profile of NCI cell line panel have been reported (Efferth and Kaina, 2004; Sertel et al., 2012). Here, we focused on the Novartis microarray platform. In all studies the chi-square test reveal a significant clustering of the cell lines based on their sensitivity or resistance toward As_2_O_3_. Moreover, several biological functions of the genes, e.g., cell cycle progression, and proliferation related genes, signal transduction and apoptosis-regulating gene were common in the previous and the present investigations.

Interestingly, the transcription factors binding motif analysis revealed two transcription factors (NF-κB and AP-1) which are closely connected not only to carcinogenesis but also with drug resistance (Boeckx et al., 2015). Therefore, we evaluated the potential of As_2_O_3_ to inhibit the activity of both AP-1 and NF-κB. NF-κB and AP-1 regulate numerous important cellular processes. NF-κB activity is regulated by IκB kinase, which phosphorylates the NF-κB inhibitor IκB. Fos is key constituent of AP-1 and is regulated by serum responsive factors (SRFs) as well as ternary complex factors (TCFs). AP-1 regulates cellular growth, differentiation, program cell death, transformation and invasion (McDonnell et al., 1990; Szabo et al., 1991; Brown et al., 1993). AP-1 complexes, particularly those that contain c-Jun, achieve their growth promoting function via repression of tumor suppressor genes, such as p53, p21cip1/waf1 and p16 (Shaulian and Karin, 2001). Although NF-κB and AP-1 are two distinct regulatory mechanisms, they may modulate each other (Fujioka et al., 2004).

Our results that As_2_O_3_ inhibited the DNA-binding activity in a reporter cell model are in accordance with other reports demonstrating that As_2_O_3_ inhibited NF-κB activity (Han et al., 2005; Wei et al., 2005; Mathieu and Besancon, 2006; Ghaffari et al., 2016). The ectopic expression of NF-κB p65 protected cells from As_2_O_3_ induced apoptosis (Ghaffari et al., 2016), indicating that the NF-κB pathway mediates resistance to As_2_O_3_.

Activator protein 1 (AP-1) is a transcription factor belongs to the group of *trans*-acting elements that consist of several proteins, including JUN, FOS, ATF (activating transcription factor) and MAF (musculoaponeurotic fibrosarcoma). AP-1 forms homodimers or heterodimers via its leucine-zipper domains, different dimerization ultimately allow that AP-1 recognizes different sequences in the promoters and enhancers of target genes (Eferl and Wagner, 2003). The AP-1 activity was causatively linked to cell transformation and it assumed to be a significant factor for tumorigenesis (Kharman-Biz et al., 2013). Therefore, AP-1 might provide opportunities for the development of novel targeted cancer treatment strategies (Angel and Karin, 1991; Milde-Langosch et al., 2004; Kharman-Biz et al., 2013). Interestingly, AP-1 drives not only carcinogenesis, but also increases the expression of multidrug resistance-conferring *MDR1* gene (Daschner et al., 1999). Therefore, c-Jun is a potential drug target to combat MDR (Passegue and Wagner, 2000).

Using an AP-1-specific reporter cell line, we found that As_2_O_3_ inhibited AP-1 activity. This result fits to data of (Yedjou and Tchounwou, 2009), who observed that the As_2_O_3_-induced cell cycle arrest was correlated with repression of c-Fos (as component of the AP-1 heterodimeric complex). In another study, As_2_O_3_ treatment also downregulated c-Fos expression (Walker et al., 2016). The second partner of AP-1, c-Jun, was more phosphorylated is As_2_O_3_-resistant cells and hypophosphorylated in As_2_O_3_-sensitive cells upon As_2_O_3_ treatment (Roszak et al., 2013).

Nuclear factor (NF-κB) is another transcription factor responsible for expression regulation of genes involve in apoptosis, drug resistance, innate and adaptive immune responses, proliferation and metastasis (Guttridge et al., 1999). It exists as inactive form in the cytoplasm by interacting with inhibitory proteins (IκB). Phosphorylation of IκB by IκB kinase (IKK) activates NF-κB. Then, NF-κB is translocated to the nucleus, where it binds to specific gene promoters to *trans*-activate the expression of target genes (Baud and Karin, 2009). NF-κB is constitutively active in hematologic malignancies and various types of solid tumors (Bharti et al., 2003). Besides, cancer cells become resistant to antitumor drugs and irradiations by NF-κB activation (Baud and Karin, 2009). Furthermore, decreased expression of anti-apoptotic genes is correlated with inhibition of NF-κB (Lin et al., 2010). Taken together the inhibition of NF-κB represents a promising target for cancer prevention and therapy (Nakanishi and Toi, 2005; Lin et al., 2010).

Several studies described that As_2_O_3_ exerts its anti-tumor activity by inhibiting AP-1 (Um et al., 2002; Brown et al., 2007). However, other studies showed that As_2_O_3_ stimulated AP-1 activity in cultured human fibroblasts and APL (Burnichon et al., 2003; Jang et al., 2012). This contradiction points to the fact that many arsenic compounds reveal both anti-cancer and pro-cancer activates. In this study, we used HEK293 cell lines transfected with AP-1 luciferase reporter construct to detect the activity of AP-1 after As_2_O_3_ treatment. We showed that As_2_O_3_ inhibit AP-1 in dose- dependent manner and this in line with previous study in HeLa, CaSki, and C33A cells (Wang et al., 2014).

On the other hand, many suggestions have been put forth on the mechanisms, by which As_2_O_3_ cause apoptosis. One of the proposed explanations is that As_2_O_3_ inhibits the NF-κB activity. It induces genes involve in cell survival, tumor growth, blocking apoptosis and metastasis (Berman et al., 2002). NF-κB up-regulates both intracellular adhesion molecules and vascular cell adhesion, and inhibits IL-6 secretion resulting from adhesion of multiple myeloma cells to bone marrow stroma (Miller et al., 2002).

Our results from NF-κB reporter assays are in line with other studies demonstrating that As_2_O_3_ significantly inhibits NF-κB activity in a dose- dependent manner. In HL-60 cells, As_2_O_3_ suppressed the DNA-binding activity of the NF-κB heterodimer (p65/p50) through inhibiting the degradation of IκB-α and then preventing nuclear translocation of p65 and the binding of NF-κB with their target gene consensus sequences (Han et al., 2005).

Although it is well known that natural products act rather in a multi-specific manner than mono-specifically against one single target (Efferth and Koch, 2011), it turns out more and more that synthetic drugs and even targeted small molecules affect multiple targets and pathways in diseased cells (Geromichalos et al., 2016). Therefore, network pharmacological approaches have been suggested to provide a more comprising picture of what is happening in cell upon drug treatment (Poornima et al., 2016). Hence, network pharmacology represents a valuable tool to investigate the mode of action of both synthetic and natural compounds. A conclusion that can be drawn from our network analysis of As_2_O_3_ is that rather entire gene clusters belonging to specific pathways and functional groups than single genes were involved in drug action. Given the individual differences of organisms and the biological flexibility to adapt to xenobiotic exposures, it is reasonable from an evolutionary point of view to assume cellular and molecular reactions of high flexibility and plasticity. The concept of the past (“one drug – one target”) obviously needs to be revisited and enlarged by a “one drug – multiple pathway” hypothesis.

The international sequencing projects (e.g., Cancer Genome Atlas,) unraveled a huge diversity of mutational profiles and gene expression patterns in individual tumors (Chin and Gray, 2008). Hence, it is reasonable that each patient’s tumor might react in a different manner to chemotherapy. In the case of As_2_O_3_, we found several major functional groups using manual curated network analysis to be involved in cellular responsiveness to the drug, i.e., oxidative stress response, DNA repair, regulation of cell cycle/proliferation, signal transduction, drug transporter, cytoskeletal elements, tumor suppressors/oncogenes, metabolic pathways, and apoptosis. On the other hand, we found many other important pathways using IPA network analysis, e.g., molecular mechanisms of cancer, axonal guidance signaling, signaling by Rho family GTPases, protein kinase A signaling, phospholipase signaling, actin cytoskeleton signaling etc. The manually curated and the IPA approaches revealed comparable findings for three types of pathways: signal transduction, metabolic pathways and cytoskeletal elements. Moreover, axonal guidance signaling, neuroregulin signaling and agrin interaction at neuromuscular junction pathways may demonstrate the neurotoxicity of As_2_O_3_. This is an interesting finding, since neurotoxicity is a known toxicity of arsenic (Vahidnia et al., 2007). Our IPA analysis identified three canonical pathways that might explain neurotoxic effects of As_2_O_3_. Furthermore, neurological effects of arsenic may lead to cytoskeletal framework disorganization which is a suggested mechanism of arsenic-induced neurotoxicity (Vahidnia et al., 2007). Interestingly, IPA network analysis also revealed several pathways which are directly connected with the cytoskeleton in addition to glioma signaling. It deserves further investigations, whether these pathways are also involved in the collateral sensitivity of the U87.MG. ΔEGFR glioblastoma cells to As_2_O_3_. Not surprisingly, IPA showed cell death and survival as top biological functions of the inserted genes with a *p*-value of 4.28E-09 and with 43 genes that contribute to this function.

Having the inter- and intra-tumor heterogeneity in mind, it is reasonable to assume that different tumors activate different genes belonging to the same main pathways and functional groups. The complexity of this process can be manage by network modeling, and corresponding bioinformatical tools are becoming more and more indispensable for pharmacology of both synthetic and natural drugs.

In conclusion, As_2_O_3_ was active against tumor cell lines. It showed collateral sensitivity toward U87.MG ΔEGFR. In addition to that, it inhibited AP-1 and NF-κB activity in a dose- dependent manner. COMPARE and cluster analyses using gene expression profiling of 58 NCI cell lines predicted the sensitivity or resistance of cancer cells to As_2_O_3_. Using a network pharmacological approach, we identified functional clusters of genes involved in the mechanisms of action in cancer cells.

## Author Contributions

MD performed the cytotoxicity, COMPARE and hierarchical cluster analyses, promoter binding motif analysis. NF-KB and AP-1 reporter assays, Interactome network analysis, and drafted the paper. SH performed the cytotoxicity and drafted the paper. TE supervised the project and corrected the paper.

## Conflict of Interest Statement

The authors declare that the research was conducted in the absence of any commercial or financial relationships that could be construed as a potential conflict of interest.

## Footnotes

1 http://dtp.nci.nih.gov

2 http://dtp.cancer.gov/databases_tools/default.htm

3 http://dtp.nci.nih.gov

4 https://discover.nci.nih.gov/cimminer/oneMatrix.do

5 http://genome.ucsc.edu

## Abbreviations

APL: acute promyelocytic leukemia
AP-1: activator protein 1
As_2_O_3_: arsenic trioxide
EGFR: epidermal growth factor receptor
NF-κB: nuclear factor-kappa in B cells
PMA: phorbol 12-myristate 13-acetate
PML: promyelocytic leukemia
RARA: retinoic acid receptor alpha
SEAP: secreted embryonic alkaline phosphatase
TNF: tumor necrosis factor
